# Prevalence and clinical characteristics of impulse control disorder in Southern Brazilian Parkinson's disease patients

**DOI:** 10.1055/s-0046-1820529

**Published:** 2026-05-18

**Authors:** Eurípedes Gomes de Carvalho Neto, Nayron Medeiros Soares, Gabriela Magalhães Pereira, Bárbara Maldotti Dalla Corte, Ana Carolina Leonardi Dutra, Nathalie Ribeiro Artigas, Rosa Maria Martins de Almeida, Júlia Schneider Krimberg, Bruno Elkfury Monticelli, Artur Francisco Schumacher Schuh, Carlos Roberto de Mello Rieder

**Affiliations:** 1 Universidade Federal do Rio Grande do Sul, Faculdade de Medicina, Porto Alegre RS, Brazil.; 2 Irmandade da Santa Casa de Misericórdia de Porto Alegre, Serviço de Neurologia, Porto Alegre RS, Brazil.; 3 Hospital de Clínicas de Porto Alegre, Serviço de Neurologia, Porto Alegre RS, Brazil.; 4 Universidade Federal do Rio Grande do Sul, Instituto de Psicologia, Porto Alegre RS, Brazil.; 5 University of Toronto, Department of Cell and Systems Biology, Toronto ON, Canada.

**Keywords:** Parkinson Disease, Disruptive, Impulse Control, and Conduct Disorders, Dopamine Agonists, Levodopa

## Abstract

**Background:**

Impulse control disorders (ICDs) are potentially serious complications of Parkinson's disease (PD). Treatment, particularly the use of dopamine agonists (DAs), is associated with the development of ICDs and related behaviors. However, susceptibility to these disorders appears to be linked to specific risk factors.

**Objective:**

To assess the frequency, clinical presentation, and factors associated with the development of ICDs in a population of patients with PD.

**Methods:**

Patients with PD were screened for ICD-related symptoms using the Questionnaire for Impulsive-Compulsive Disorders in Parkinson''s Disease -Current Short (QUIP-CS) questionnaire. Additionally, they underwent cognitive evaluation and were assessed using the Movement Disorder Society–Unified Parkinson's Disease Rating Scale (MDS-UPDRS). Sociodemographic data and disease progression information were collected. Levodopa equivalent daily dose (LEDD) was calculated for each patient.

**Results:**

Of the 90 patients evaluated, 42 (46.6%) exhibited symptoms of ICDs. The most frequent subtype was binge eating (50%), followed by compulsive buying (33.3%) and hypersexuality (21.4%). A significant association was found between DA use and the development of ICDs ( *p* = 0.041). Patients with ICDs using DAs had a higher mean LEDD ( *p* < 0.001) and a higher frequency of motor complications (MDS-UPDRS Part IV, *p* = 0.028) compared to those not using DAs.

**Conclusion:**

In the current study, the use of DAs was the main risk factor associated with the development of impulse control disorders. No other significant associated factors could be identified.

## INTRODUCTION

In the treatment of Parkinson's disease (PD), the chronic use of dopaminergic medications has been associated with motor and non-motor side effects, such as dyskinesias and impulse control disorders (ICDs). 1 The latter correspond to a heterogeneous group of disorders that involve pleasurable behaviors performed repetitively, excessively, and compulsively. 2 The most common types exhibited by patients with PD include pathological gambling, binge eating, hypersexuality, and compulsive buying. 3 These behavioral disturbances can present in several degrees of severity, leading to functional impairment and reduced quality of life 4 and, in some cases, to devastating financial, legal, and psychosocial consequences. 5

The prevalence of ICDs varies across studies: 13.6% in the DOMINION study (United States/Canada), 6 29.3% in ICARUS (Italy), 7 10.1% in South Korea, 8 and 58.3% in Spain. 9 In Brazil, Valença et al. 10 found an ICD prevalence of 18.4% in patients from the Northeast. No studies have evaluated ICDs specifically in Southern Brazil, a region with distinct cultural and socioeconomic factors.

The aim of the present study is to analyze the presence of ICDs in a sample of patients with PD in southern Brazil. We hypothesized that the frequency and subtype distribution of ICDs may differ regionally, and that the use of dopamine agonists (DA) would be associated with these disorders, which is consistent with international findings. This is the first analysis of ICDs and its associated factors in a PD population from southern Brazil, providing relevant epidemiological data for a region that has not been previously studied.

## METHODS

### Study design

This research was approved by the research ethics committee of the Hospital de Clínicas de Porto Alegre (HCPA), Porto Alegre, Rio Grande do Sul, Brazil, under protocol no. 2.106.230, and was conducted in accordance with the Declaration of Helsinki.

The present was an observational, cross-sectional, analytic study and followed the Strengthening the Reporting of Observational Studies in Epidemiology (STROBE) guidelines. The study was funded by Coordenação de Aperfeiçoamento de Pessoal de Nível Superior (CAPES) and by the Hospital de Clínicas de Porto Alegre (HCPA)/Fundo de Incentivo à Pesquisa e Eventos (FIPE).

### Participants

Patients were recruited from the movement disorders outpatient clinic of the HCPA Neurology Service. A total of 103 patients diagnosed with PD were initially screened. Diagnosis was established by movement disorders specialists using the UK Brain Bank criteria. Recruitment was consecutive in the movement disorders outpatient clinic.

Data collection occurred from May 2017 to February 2020. All participants provided written consent after an explanation of the study procedures. Following consent, participants underwent a single individual interview by a trained researcher, who administered the assessment scales and questionnaires.

Participants were required to meet the following criteria: absence of severe cognitive impairment, defined as Montreal Cognitive Assessment (MoCA) < 18 or inability to complete testing; Hoehn and Yahr stages I to IV; no history of chemical or alcohol dependence; no major psychiatric disorders (bipolar disorder, psychosis, schizophrenia-spectrum disorders); and no prior deep brain stimulator (DBS) implantation.

### Evaluation

Participants completed a sociodemographic questionnaire that collect data on age, education, sex, and marital status. Additional clinical data included disease duration, age at symptom onset, antiparkinsonian medications in use, and their respective dosages. The levodopa equivalent daily dose (LEDD) was also calculated for each patient.

The presence of symptoms related to ICDs were assessed by the validated Brazilian Portuguese version of the Questionnaire for Impulsive-Compulsive Disorders in Parkinson's Disease (QUIP), “current short” version, which evaluates symptoms present at the time of assessment. Cognitive evaluation included the MoCA, the semantic verbal fluency test (animal category) and the phonemic verbal fluency test (letters F-A-S). All patients were further evaluated using the Movement Disorder Society – Unified Parkinson's Disease Rating Scale (MDS-UPDRS).

### Statistical analysis

To assess the normality of data distribution, the Shapiro-Wilk test was applied. Groups were compared using the unpaired t-test, Mann-Whitney test, or the chi-squared test, according to distributions of the variables. The results were expressed as absolute frequencies, mean and standard deviation (SD), or median and interquartile range (IQR, upper–lower quartiles) values, with 95%CIs. A linear regression model was performed, adjusted for monoamine oxidase inhibitors (MAOIs), catechol-o-methyl-transferase inhibitors (COMTIs), and amantadine, or adjusted by sex and age. For all analyses, statistical significance was considered *p* < 0.05. Statistical analysis was performed using the IBM SPSS Statistics for Windows (IBM Corp.) software, version 24.0.

## RESULTS

A total of 13 patients (12.6%) excluded according to the exclusion criteria: severe cognitive impairment (MoCA < 18: n = 7); substance dependence (n = 3); DBS implant (n = 2); and major psychiatric comorbidity (bipolar disorder: n = 1).

The final sample consisted of 90 patients with PD. Of those, 42 patients (46.6%) showed symptoms consistent with ICDs based on the QUIP. Male prevalence was similar between groups: 52.38% in the group with ICD-positive group and 54.16% in the ICD-negative group. The most frequent ICD subtype in our sample was binge eating (50%), followed by compulsive buying (33.33%), and hypersexuality (21.42%). Notably, pathological gambling was observed in only one patient (2.4% of ICD-positive individuals). Dopaminergic dysregulation syndrome, related to the excessive use of dopaminergic drugs corresponded to 7.14% of the cases. Additionally, 57.1% of the patients fell in the “other behaviors” category, which includes hobbyism, punding, and walkabout.

In Table 1 , patients were categorized according to the presence (positive QUIP) or absence (negative QUIP) of symptoms related to ICD. The two groups were compared with respect to age, education, sex, disease duration, age at symptom onset, living arrangement, smoking or coffee consumption, MDS-UPDRS score, use of DAs, MAOIs and COMTIs, amantadine use, and LEDD. Cognitive performance was also compared between groups based on MoCA, phonemic fluency (FAS) scores, and semantic fluency (animal F-A-S).

**Table 1:** Descriptive of patients with and without impulse control disorder (n = 90)

Variables		Positive QUIP (n = 42)	Negative QUIP (n = 48)	*p-* value
Age (years)		63.85 (39.69–77.47)	63.29 (43.01–78.47)	0.815
Years of schooling		9.00 (2.00–27.00)	11.00 (3.00–22.00)	0.874
Gender (M/F)		22/20	26/22	1.000
Disease duration (years)		9.00 (1.00–21.00)	9.00 (2.00–29.00)	0.836
Age at symptom onset (years)		51.00 (25.00–69.00)	52.00 (33.00–66.00)	0.768
Living arrangement	Alone	13	15	1.000
	With others	29	33	
MDS-UPDRS	1	15.00 (3.00–34.00)	14.00 (3.00–39.00)	0.694
	2	17.00 (4.00–37.00)	19.00 (0.00–48.00)	0.512
	3	43.43 ± 13.52	45.46 ± 17.92	0.550
	4	4.50 (0.00–19.00)	5.00 (0.00–21.00)	0.548
MDS-UPDRS Total		82.21 ± 28.19	87.06 ± 33.15	0.460
Smoking habit (Y/N)		9/30	16/27	0.251
Coffee consumption (Y/N)		34/8	34/12	0.594
QUIP	Pathological gambling (Y/N)	1/41	**-**	−
	Hypersexuality (Y/N)	9/33	**-**	−
	Compulsive buying (Y/N)	14/28	**-**	−
	Binge eating (Y/N)	21/21	**-**	−
	Other behaviors (Y/N)	24/18	**-**	−
	Dopamine dysregulation syndrome (Y/N)	3/39	**-**	−
Dopaminergic agonist (Y/N)		23/19	15/33	0.041*
MAOIs (Y/N)		2/40	6/42	0.276
COMTIs (Y/N)		5/37	2/46	0.245
Amantadine (Y/N)		17/25	18/30	0.942
LEDD		1,075.00 (237.50–2,928.00)	1,100.00 (400.00–4,150.00)	0.974
MoCA total		26.50 (13.00–30.00)	25.00 (12.00–30.00)	0.521
FAS animals		16.61 ± 5.29	16.63 ± 6.03	0.687
FAS		34.00 (3.00–70.00)	36.00 (0.00–78.00)	0.566
FAS total		48.02 ± 18.41	53.41 ± 22.60	0.237

Abbreviations: COMTIs, catechol-O-methyltransferase inhibitors; F, female; FAS animals, semantic verbal fluency test category – animals; FAS, phonemic verbal fluency test; LEDD, Levodopa equivalent daily dose; M, male; MAOIs, monoamine oxidase inhibitors; MDS-UPDRS, Movement Disorder Society-Sponsored Revision of the Unified Parkinson's Disease Rating Scale; MoCA, Montreal Cognitive Assessment; N, no; QUIP, Questionnaire for Impulsive-Compulsive Disorders in Parkinson''s Disease; Y, yes.

A significant association was found between the use of DAs and the presence of ICDs (positive QUIP; *p* = 0.041). In our sample, 54% (n = 23) of the patients who had symptoms of ICDs were using a DA, whereas only 31.25% (n = 15) without symptoms were using this class of medications. No significant differences were observed between the groups regarding age, education, gender, time of diagnosis, age at symptom onset, living with a partner or not, smoking or coffee consumption habits, MDS-UPDRS scores, use of MAOIs, COMTIs, amantadine, or LEDD. Likewise, no significant differences were found in cognitive assessment scores between the two groups.

Table 2 includes all the patients with ICDs (symptoms verified using the QUIP). These patients were categorized into two groups: those using agonists and those not using the medication. The two groups were compared with respect to age, education, gender, time since diagnosis, age at symptom onset, living arrangement, smoking or coffee consumption habits, MDS-UPDRS scores, use of MAOIs, COMTIs, amantadine, and LEDD. Cognitive performance was also compared using MoCA, phonemic fluency (FAS), semantic fluency (animals), and total FAS scores.

**Table 2:** Descriptive of patients with impulse disorder (n = 42)

Variables		Dopaminergic agonist (n = 23)	No dopaminergic agonist (n = 19)	*p-* value
Age (years)		62.00 (39.69–71.58)	68.82 (40.20–77.47)	0.079
Years of schooling		10.8 ± 6.59	9.5 ± 4.32	0.447
Gender (M/F)		12/11	9/10	1.000
Disease duration (years)		10.39 ± 4.54	8.74 ± 4.86	0.262
Age at symptom onset (years)		49.00 (25.00–64.00)	56.00 (32.00–69.00)	0.095
Living arrangement	Alone	7	6	1.000
	With others	16	13	
MDS-UPDRS	1	16.65 ± 8.04	12.58 ± 6.04	0.076
	2	19.52 ± 10.43	17.11 ± 8.31	0.419
	3	42.30 ± 13.31	44.79 ± 14.01	0.560
	4	8.00 (0.00–19.00)	2.00 (0.00–15.00)	0.028*
MDS-UPDRS Total		85.74 ± 30.44	77.95 ± 25.35	0.379
Smoking habit (Y/N)		4/17	5/13	0.706
Coffee consumption (Y/N)		19/4	15/4	1.000
QUIP	Pathological gambling (Y/N)	0/23	1/18	0.452
	Hypersexuality (Y/N)	6/17	3/16	0.477
	Compulsive buying (Y/N)	8/15	6/13	1.000
	Binge eating (Y/N)	12/11	9/10	1.000
	Other behaviours (Y/N)	15/8	9/10	0.395
	Dopamine dysregulation syndrome (Y/N)	2/21	1/18	1.000
MAOIs (Y/N)		1/22	1/18	1.000
COMTIs (Y/N)		4/19	1/18	0.356
Amantadine (Y/N)		11/12	6/13	0.452
LEDD		1,444.46 ± 660.27	831.18 ± 406.38	0.001*
MoCA total		25.00 (13.00–29.00)	27.00 (13.00–30.00)	0.387
FAS animals		15.40 ± 4.98	16.89 ± 5.63	0.386
FAS		36.50 (3.00–51.00)	32.00 (14.00–70.00)	0.607
FAS total		52.50 (11.00–69.00)	46.00 (23.00–97.00)	0.989

Abbreviations: COMTIs, catechol-O-methyltransferase inhibitors; F, female; FAS animals, semantic verbal fluency test category animals; FAS, phonemic verbal fluency test; LEDD, Levodopa equivalent daily dose; M, male; MAOIs, monoamine oxidase inhibitors; MDS-UPDRS, Movement Disorder Society-Sponsored Revision of the Unified Parkinson's Disease Rating Scale; MoCA, Montreal Cognitive Assessment; N, no; QUIP, Questionnaire for Impulsive-Compulsive Disorders in Parkinson's Disease; Y, yes.

The ICD group using a DA had a significantly higher median score on part IV of the MDS-UPDRS ( *p* = 0.028), indicating greater motor complications and dyskinesias compared with ICD patients who were not using DAs. As expected, this group also had a higher levodopa-equivalent daily dose (LEDD, *p* = 0.001). No other variants showed statistically significant difference between the two groups.

A linear regression model was performed, adjusted for MAOIs, COMTIs, and amantadine, or for gender and age. In this model, for each 1 mg increase in pramipexole dose, an increase of 0.32 and 0.35 points in the QUIP score was observed ( Table 3 ).

**Table 3:** Total QUIP linear regression

Pramipexol	B	*p-* value
Adjusted by MAOIs, COMTIs, and amantadine	0.350	0.004
Adjusted for gender and age	0.326	0.009

Abbreviations: COMTIs, catechol-O-methyltransferase inhibitors; MAOIs, monoamine oxidase inhibitors; QUIP, Questionnaire for Impulsive-Compulsive Disorders in Parkinson's Disease.

## DISCUSSION

The current study showed a high percentage of patients with symptoms compatible with ICDs (46.6% of the sample). In the literature, there is substantial variability in the reported prevalence of ICDs and their subtypes ( Table 4 ).

**Table 4:** Comparison of studies on ICD in patients with Parkinson's disease

Author (year)	Study location	N	ICD screening instrument	ICDs (%)	ICD subtype (%)	Associated factors
Weintraub et al. (2010) 6	United States and Canada	3090	MIDI	13.6%	GD: 5 HS: 3.5 CB: 5.7 BE: 4.3 MICD: 3.9	Dopamine agonist use; levodopa use; living in the United States; younger age; being unmarried; current cigarette smoking; family history of gambling problems.
Antonini et al. (2017) 7	Italy	1069	MIDI and QUIP	29.3	GD: 5.4 HS: 14.6 CB: 6.1 BE: 11.4 PRB: hobbyism (12.1) Punding (7) Walkabout (1.3) DDS: 3.5	Male; younger; younger at PD onset; longer disease duration; more severe non-motor symptoms (including mood and sexual function); depressive symptoms; sleep impairment; and poorer PD-related QoL.
Lee et al. (2010) 8	South Korea	1167	MIDI	10.1	GD: 1.3 HS: 2.8 CB: 2.5 BE 3.4 MICD: 2.91 PRB: 4.2	Dose response relationships between the dopamine agonist dose and the ORs for compulsive buying, gambling and sexual behaviors. OR for punding was positively correlated with the dose of levodopa. OR for compulsive eating was not associated with the dose of dopamine agonist or levodopa.
Vela et al. (2016) 9	Spain	87	QUIP	58.3	GD: 10.7 HS: 23.8 CB: 15.5 BE: 20.2 PRB: Hobbyism (29.8) Punding (17.9)	DA intake. ICD patients had a higher frequency of motor fluctuations, a higher score in the BDI and a worse QoL.
El Otmani et al. (2019) 11	Morocco	125	QUIP	28	GD: 3.2 HS: 8 CB: 9.6 BE: 7.2 MICD: 14 PRB: 11.1	Young age; male gender; and dopamine agonist equivalent daily dose.
Valença et al. (2013) 10	Brazil	152	QUIP	18.4	GD: 1.3 HS: 11.8 CB: 10.5 BE: 7.9 PRB: 14.2 DDS: 0.65	Dopamine agonist use and previous smoking.
Present Study	Brazil	90	QUIP	46.6	GD: 1.1 HS: 10 CB: 15.5 BE: 23.3 MICD: 23.3 PRB: 26.6 DDS: 3.3	Dopamine agonist use.

Abbreviations: BDI, Beck depression inventory; BE, compulsive binge eating disorder; CB, compulsive buying disorder; DDS, dopamine dysregulation syndrome; GD, gambling disorder; HS, hypersexuality; ICDs, Impulse control disorders; MICD, Multiple impulse control disorder; MIDI, Minnesota Impulsive Disorders Interview; OR, odds ratio; PD, Parkinson's disease; PRB, Punding and related behaviors; QUIP, Questionnaire for Impulsive-Compulsive Disorders in Parkinson's Disease; QoL, Quality of Life.

A Spanish study found a prevalence of 58.3%. 9 The DOMINION study, a large multicenter, cross-sectional investigation, found about 13.6% of patients with ICDs. 6 The ICARUS study in Italy found ICD symptoms in 29.3% of patients at 1-year of follow-up. 7 A study from Morocco reported a similar percentage, with approximately 28% of patients with ICDs. 11 In South Korea, a study showed only 10.1%. 8 In Brazil, a study conducted in Bahia investigated 152 patients with PD and found a prevalence of 18.4% of patients with ICDs. 10

In the present study, the most frequent ICD subtype was binge eating, followed by compulsive buying and hypersexuality. In the ICARUS study, binge eating was also the most prevalent subtype, followed by hypersexuality. 7 The Korean study similarly reported binge eating followed by hypersexuality as the most prevalent subtypes. 8 In the DOMINION study, the most frequent subtypes were compulsive buying and pathological gambling. 6 In Morocco, compulsive buying was also the most common subtype, followed by hypersexuality. 11 In the Bahia study, hypersexuality and compulsive buying were the most prevalent behaviours. 10 In contrast, the Spanish study identified hobbyism as the most prevalent subtype. 9

In our sample, pathological gambling was identified in only one patient, representing a remarkably low proportion compared to reports from North American and European cohorts, where gambling behaviors are frequently reported among the most common ICD subtypes. This finding likely reflects sociocultural characteristics of southern Brazil, where access to legalized gambling is limited and gambling is less culturally prevalent than in regions where such behaviors are studied more extensively.

Furthermore, we found that 57.1% of the patients with ICDs (23.3% of the total sample) were in the “other behaviors” category, which includes hobbyism, punding, and walkabout. In the literature, punding is estimated to affect approximately 0.34 to 14% of patients with PD. 12 The wide variability in the prevalence of ICDs and their subtypes is influenced not only by cultural factors (e.g., the higher frequency of gambling disorder in the United States) but also by methodological differences across studies, including sampling strategies, data collection procedures, screening instruments used, among other factors. 13

Regarding dopaminergic dysregulation syndrome, prevalence in a systematic review ranged from 0.6 to 7.7%. 14 In our sample, it accounted for 7.14% of ICD cases (3.3% of the total PD sample).

In the present study, we compared patients with ICDs who were using DAs with those who were not. The aim was to assess whether there were distinct clinical characteristics between these groups. There was a statistically significant difference ( *p* < 0.001) in LEDD between groups: the group using DAs had an average of 1,444.46 ± 660.27, whereas the group not using DA had an average of 831.18 ± 406.38. A higher frequency of motor complications (assessed through part IV of the MDS-UPDRS) was also observed in the ICD patients using DAs ( *p* = 0.028). These findings likely reflect treatment strategies, since DAs are often used as part of the management of motor fluctuations and dyskinesias.

Some studies have reported an association between ICDs and the use of MAOIs, COMTIs, and amantadine. 6 15 16 However, in the current study, no such association was observed. On the other hand, consistent with several previous studies, the use of DAs was associated with the development of ICDs ( Table 1 ). 8 17

Early onset of PD motor symptoms and not being married have been described in some studies as additional associated factors. 6 7 9 In our sample, marital status and age at symptom onset were not associated with the occurrence of ICDs.

Regarding cognitive assessment, some studies have reported that patients with ICDs perform worse on tasks assessing executive functions of the frontal lobes, 18 such as the Trail Making test. These tests would assess cortical areas that are more vulnerable to the influence of dopamine levels (e.g., the ventromedial and orbitofrontal cortices) and, therefore, more susceptible to the effects of dopaminergic medications. 18 19 In the current study, the MoCA was used. Although it is a more general cognitive screening instrument, it includes an assessment of executive function. The comparison of MoCA scores between groups with and without ICD showed no statistically significant difference. Additionally, other studies have suggested that patients with ICDs would perform better on tasks related to verbal and semantic fluency. 19 In the present study, there were no differences between groups on these tests either.

In conclusion, the use of DAs remains the main risk factor associated with the development of ICDs in our setting. Furthermore, the influence of other factors beyond dopaminergic therapy on the development of ICDs was not demonstrated.

We also sought to determine whether patients with ICDs who use DAs differ clinically from those who do not. The only difference identified was in UPDRS part IV scores. However, this finding is likely attributable to treatment strategies, since DAs are commonly used in the management of dyskinesias.

The current study has some limitations, including the absence of a non-PD control group. Additionally, the sample is relatively small and represents only a subset of the PD population in southern Brazil, which restricts the generalizability of the findings to the broader national context. Nonetheless, the sample size is comparable to that of other international studies investigating ICDs and reflects the patient profile of a major tertiary referral center in the region.

Despite these limitations, the study is relevant, as few investigations have examined factors related to the development of ICDs in Brazilian populations. Larger and longitudinal studies will be essential to further elucidate the determinants and trajectory of ICDs in our setting.
